# Adding a dimension to cell fate

**DOI:** 10.21451/1984-3143-AR2018-0096

**Published:** 2020-05-22

**Authors:** Tiziana A.L. Brevini, Elena F.M. Manzoni, Sharon Arcuri, Fulvio Gandolfi

**Affiliations:** Department of Health, Animal Science and Food Safety, Università degli Studi di Milano, Milano 20122, Italy.

**Keywords:** 3D culture, mechano-sensing, epigenetic conversion

## Abstract

Cell fate specification, gene expression and spatial restriction are process finely tuned by epigenetic regulatory mechanisms. At the same time, mechanical forces have been shown to be crucial to drive cell plasticity and boost differentiation. Indeed, several studies have demonstrated that transitions along different specification states are strongly influenced by 3D rearrangement and mechanical properties of the surrounding microenvironment, that can modulate both cell potency and differentiation, through the activation of specific mechanosensing-related pathways. An overview of small molecule ability to modulate cell plasticity and define cell fate is here presented and results, showing the possibility to erase the epigenetic signature of adult dermal fibroblasts and convert them into insulin-producing cells (EpiCC) are described. The beneficial effects exerted on such processes, when cells are homed on an adequate substrate, that shows “*in vivo”* tissue-like stiffness are also discussed and the contribution of the Hippo signalling mechano-transduction pathway as one of the mechanisms involved is examined. In addition, results obtained using a genetically modified fibroblast cell line, expressing the enhanced green fluorescent protein (eGFP) under the control of the porcine insulin gene (INS) promoter (INS-eGFP transgenic pigs), are reported. This model offers the advantage to monitor the progression of cell conversion in real time mode. All these observations have a main role in order to allow a swift scale-up culture procedure, essential for cell therapy and tissue engineering applied to human regenerative medicine, and fundamental to ensure an efficient translation process from the results obtained at the laboratory bench to the patient bedside. Moreover, the creation of reliable in vitro model represents a key point to ensure the development of more physiological models that, in turn, may reduce the number of animals used, implementing non-invasive investigations and animal welfare and protection.

## Introduction

Epigenetic mechanisms play a key role in cell fate specification and ensure a proper regulation of gene expression and cell spatial restriction. Several studies have demonstrated the possibility to revert differentiation process, reactivating silenced genes (Palii *et al*., 2008) and facilitating cell transition to a different lineage (Taylor *et al*., 1984). Beside the epigenetic mechanisms driving cell conversion processes, growing evidences highlight the importance of mechanical forces in supporting cell plasticity and boosting differentiation. These transitions along different specification states are strongly influenced by 3D rearrangement and mechanical properties of the cellular microenvironment, that affect both cell potency and differentiation, through the involvement of specific mechanosensing-related pathways. 

In this manuscript, the ability of small molecules to modulate cell plasticity and define cell fate is summarized, describing epigenetic erasing and conversion of dermal fibroblasts into insulin-producing cells (EpiCC). Furthermore, the beneficial effects exerted on these processes by the use of an adequate substrate, that displays “*in vivo”* tissue-like stiffness is discussed. Moreover, the results obtained from the conversion of a genetically modified cell line that expresses the enhanced green fluorescent protein (eGFP) under the control of the porcine insulin gene (INS) promoter (INS-eGFP transgenic pigs) are presented. Finally, in order to better characterize the mechanisms involved, the contribution of the Hippo signalling mechano-transduction pathway along the processes are examined.

## Epigenetic strategies to erase and rewind cell fate

The mammalian body is composed by more than 200 types of cells, each of these arises from the zygote, a single cell with half-genome from each parent. During embryonic development, pluripotent cells progressively restrict their ability to adopt multiple lineages to ultimately give rise to a wide variety of specialized cells. The process is driven by several factors, both extrinsic and intrinsic to the cell (Swain *et al*., 2002), that induce differential gene expression and epigenetic restrictions. Cell commitment and differentiation are spatially and temporally regulated and they occur without any permanent loss or alteration of genetic material, but rather through modifications “on top of it”. These changes are defined as epigenetic modifications and regulate the accessibility to transcription factors, in either a positive or a negative manner. They are responsible for the ‘epigenetic memory’ that underlies the phenotypic stability of the differentiated cell state, during subsequent cell divisions (Zhu *et al*., 2013; Jost, 2014; Shipony *et al*., 2014; Brevini *et al*., 2015). However, the differentiation process is reversible and may be altered by biochemical and biological manipulations, making it an attractive target to reactivate hypermethylated genes and facilitate cell phenotype changes. During the last years, the possibility to interact with the epigenetic signature of a terminally differentiated cell, switching its original phenotype into a different one, has been extensively described (Agostini *et al*., 1999; Yoshida *et al*., 2009; Brevini *et al*., 2014; Pennarossa *et al*., 2014; Mirakhori *et al*., 2015; Tan *et al*., 2015; Brevini *et al*., 2016; Chandrakanthan *et al*., 2016). In particular, it has been demonstrated that a short exposure to a demethylating agent is sufﬁcient to erase the cell original phenotype and induce in terminal differentiated somatic cells a high plasticity state. In our experiments, we selected, among the many epigenetic erasers available, 5-azacytidine (5-aza-CR), a DNA methyltransferase (DNMT) inhibitor known to activate the expression of silent genes (Jones, 1985) and to alter the differentiation state of embryonic (Constantinides *et al*., 1977) and mesenchymal cell lines (Darmon *et al*., 1984). These events have been shown to be related to a direct ten-eleven translocation 2 (TET2)-mediated demethylating effect (Manzoni *et al*., 2016) that accompanies the well-known 5-aza-CR ability to deplete DNMT 1 enzymatic activity (Christman, 2002). Indeed, TET enzymes affect cytosine methylation through an active mechanism that converts and oxidizes 5-methylcytosine (5mC) to 5-Formylcytosine (5-fC) and 5-Carboxylcytosine (5-caC), with an overall decrease of global methylation. In agreement whit this, it has been demonstrated that TET activities are indispensable for complete factor-driven reprogramming of somatic cells into iPSC.

## Epigenetic cell conversion: when you can judge a book from its cover

The high plasticity state, achieved by cells after 5-aza-CR treatment, allows a complete and direct differentiation into a new mature and functional cell type. Indeed, once cells enter into the higher plasticity window, they can easily be directed towards a different phenotype through the use of specific differentiation stimuli (Brevini *et al*., 2014; Pennarossa *et al*., 2014; Brevini *et al*., 2016). In recent paper (Pennarossa *et al*., 2013) human dermal fibroblasts derived from adult individuals were converted into insulin secreting cells using a brief exposure to 1µM 5-aza-CR immediately followed by a three-step pancreatic inducing protocol. At the end of the epigenetic conversion process, fibroblasts acquire an epithelial morphology and express the main pancreatic hormones and glucose sensor genes, distinctive of mature endocrine cells. Furthermore, 35 ± 8.9% of starting cell population is able to actively release C-peptide and insulin after exposure to 20 mM glucose, showing a dynamic response similar to pancreatic β-cells, in which changes in ambient glucose represent the primary and physiological stimulus for insulin secretion. Functionality, efficacy and safety of EpiCC have also been demonstrated *in vivo* with injection of converted cells into streptozotocin-induced diabetic mice that restored and stably maintained physiological glycemic levels after engraftment, with absence of malignant transformation and cell migration to organs and lymph nodes (Brevini *et al*., 2018). Moreover, a modified protocol that allows epigenetic conversion of fibroblasts into beta-like cells was applied to the swine, feline as well as canine species, implementing the concept that epigenetic conversion is a reproducible and robust technique, that can find useful applications in veterinary medicine and management of diabetes in pet animals (Pennarossa *et al*., 2014; Brevini *et al*., 2016).

Notably, epigenetic conversion was successfully used in different cell types, such as granulosa cells that were converted into muscle cells and human fibroblasts that were differentiated into trophoblastic-like cells (Brevini *et al*., 2014; Arcuri *et al*., 2018; Università degli Studi di Milano, Milan, Italy; unpublished data). 

## Adding a new dimension to cell fate specification

**Figure 1:**
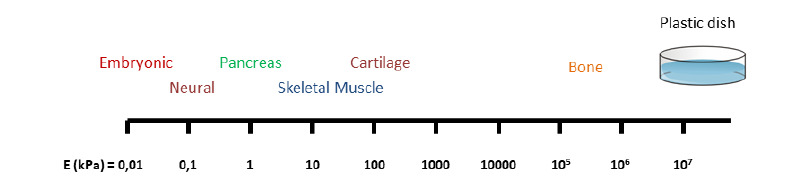
All tissues have specific biomechanical properties, expressed as stiffness (E; elastic modulus) and measured in kilopascals (kPa). These properties vary among organs and tissues, and are strictly related to tissue function.

Recent works addressed their attention to tissue architecture and mechanical forces and indicated the involvement of physical and mechanical cues (together with chemical signals) in the control of cell plasticity and differentiation. The use of 3D matrix is particularly advantageous, in this respect and allows for the production of organized arrangements of cells, displaying an architecture closer to the in vivo one. Several *in vitro* studies showed, in particular, that the use of a surface matching the stiffness of native tissues, exerts a direct effect on lineage commitment, positively influencing cell differentiation (Engler *et al*., 2006; Evans *et al*., 2009; Gilbert *et al*., 2010; Huebsch *et al*., 2010) and might be crucial for specific cellular functions (Schellenberg *et al*., 2014). In line with this, it was reported that 3D culture systems, mimicking the native tissue of embryonic stem cells (ESC), were able to exert significant eﬀects on cell shape and induced changes in chromatin structures and epigenetic remodeling, increasing cell plasticity and pluripotency (Demirkaya *et al*., 2016; Heise *et al*., 2016). The use of micro-wells and microarrays, more in details maximized cell-to-cell contact and allowed ESC to form 3D micro-aggregates with high cell density, resulting in low oxygen concentration levels that induce and promote self-renewal and pluripotency maintenance (Laschke *et al*., 2010; Kaneko *et al*., 2012; Yamamoto *et al*., 2012). All these experiments showed a strong relationship between fate commitment and mechanical cues, which was further supported by recent works, demonstrating the possibility to combine mouse ESCs and extra-embryonic trophoblast stem cells (TSCs) using a 3D scaffold that allowed to generate aggregates whose morphogenesis was remarkably similar to natural embryos (Harrison *et al*., 2017; Rivron *et al*., 2018). All these show that the microenvironment, provided by the traditional polystyrene culture systems, fails to imitate the physiological and biochemical features of cells and causes deviations in cell response. This is mainly related to the significant differences between the stiffness of the original tissue and that of several gigapascal (GPa) of the plastic support traditionally used (Fig. 1) that provides a static environment, does not allow a detailed comprehension of the natural tissue architecture, and leads to the development of a physiologically limited model. In line with this, epigenetic erasing of dermal fibroblasts and their pancreatic differentiation into insulin-producing cells was boosted by the use of soft substrate, able to mimic the in vivo pancreatic tissue stiffness (Pennarossa *et al*., 2018). In these experiments, cell mechano-sensing, and biomechanical properties of the surrounding matrix was shown to influence the acquisition of cell plasticity and enhance tissue differentiation, increase conversion efficiency and encourage the acquisition of a mature pancreatic phenotype. These data have interesting technological impacts in order to increase reliability and increment efficiency of the conversion process. This represents a key point to ensure an efficient and fast translation process from the laboratory bench to the patient bedside, since it allows a reduction in the time required from patient biopsy to the generation of a sufficient number of fully matured cells, ready for the engraftment.

## YAP and TAZ: the two main actors

**Figure 2:**
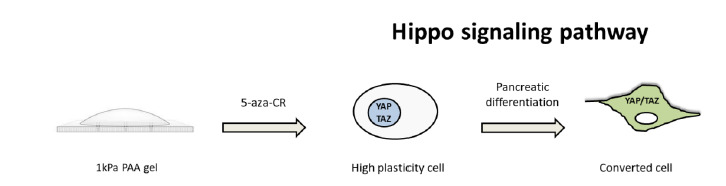
Schematic representation of the Hippo signaling pathway. Cells plated on soft polyacrylamide gel (1kPa PAA gel) and erased with 5-aza-CR enter a high plasticity state. As ESC, iPSC and any self-renewing cell type, they show YAP nuclear accumulation. At the end of the epigenetic conversion, differentiated cells exhibit nuclear exclusion and cytoplasmic confinement of YAP.

Despite increasing evidences demonstrated that cell shape, extracellular matrix (ECM) elasticity and cytoskeletal tension play important roles in cell behaviour and physiology, the way and the molecular components that perceive and transduce mechanical signals into the cells remain poorly understood. Recent studies identified yes-associated protein/WW domain containing transcription regulator 1 (YAP/TAZ), the main transcriptional effectors of the Hippo signaling pathway, as key mechano-transducers acting by nuclear relays of mechanical stimuli. In mammals, the Hippo pathway is constituted of a cascade of kinases, such as MST 1/2 and LATS 1/2, which lastly phosphorylate YAP/TAZ leading to its inactivation and exclusion for nuclear accumulation, while its dephosphorylation cause YAP/TAZ activation and translocation from the cytoplasm to the nucleus (Piccolo *et al*., 2014). Several evidences revealed the role of YAP/TAZ as main sensor for mechanical stimuli including cell density, matrix stretch and stiffness. Experiments from the Piccolo’s lab, as well as others, have examined YAP/TAZ activation after altering cellular or extracellular mechanical properties (Dupont *et al*., 2011; Wang *et al*., 2016). Moreover, cytoplasmic confinement of YAP has been reported to be distinctive of proliferative and terminally differentiated cells. In contrast high plasticity cells showed the presence of the protein in the nucleus, as well as in the cytoplasm (Hemberger *et al*., 2009; Chowdhury *et al*., 2010; Higuchi *et al*., 2014), demonstrating nuclear YAP essential role in ESC self-renewal and in the control of the levels of the pluripotency genes Oct4, Nanog and Sox2 (Lian *et al*., 2010; Young, 2011; Beyer *et al*., 2013). In line with these findings, YAP cytoplasmic accumulation was detected in differentiated EpiCC. However, those converted on soft substrate showed a significantly higher nuclear immuno-positivity exclusion compared to cells grown on plastic. Furthermore, YAP nuclear localization was described in cells exposed to 5-aza-CR regardless of the matrix elasticity selected, most likely in relation with the newly acquired high plasticity state (Fig. 2). 

Altogether these findings suggest that mechano-sensing influences the acquisition of cell plasticity and induce a significantly higher differentiation efficiency, encouraging the acquisition of a mature pancreatic phenotype. They also indicate a fundamental role of the transcriptional regulators YAP and TAZ as downstream elements in how cells receive their physical microenvironment. However, the impact of other mechanical factors on YAP/TAZ activity, as well as the involvement of others molecules in the mechano-transduction related pathway, require further investigation.

## Real time monitoring of pancreatic conversion using *INS*-eGFP fibroblasts

**Figure 3:**
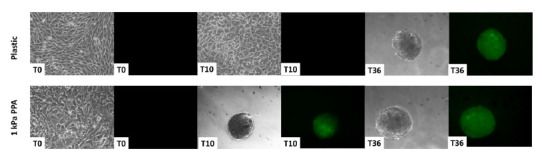
Real-time monitoring of pancreatic differentiation using INS-eGFP porcine fibroblasts plated on standard plastic dishes (plastic) and PAA gels (1kPa PAA) at different time points of the endocrine pancreatic induction protocol. When cells acquire a pancreatic phenotype and begins to produce Insulin, they emit green fluorescence, thanks to the activation of the Green Fluorescent Protein linked to the Insulin construct.

Many physiological phenomena such as cellular differentiation, proliferation and communication can be attributed to differential gene expression that is tightly regulated in response to intrinsic developmental programs and extrinsic signals. In order to obtain insightful information about the relationship between the activation/inhibition of different pathways and their effects on gene expression, specific response elements are fused to genes encoding reporter proteins. To date, reporter genes are widely used in both in vitro and in vivo applications to study the promoter and enhancer sequences, mRNA processing and translation (Jiang *et al*., 2008). Most reporter genes encoded enzymes whose activities can only be monitored by the addition of adequate substrates requiring cell lysis or fixation. The introduction of green fluorescent protein (GFP) as a fluorescent reporter (Chalfie *et al*., 1994) has improved the gene tagging approach allowing non-invasive monitoring of gene transfer and protein location in living cells. Presently, GFP is one of the most frequently used reporter genes in biological systems. Recently Wolf’s laboratory generated transgenic pigs that express enhanced GFP (eGFP) under the control of the porcine insulin gene (*INS*) promoter (*INS*-eGFP transgenic pigs) to facilitate the identification and isolation of porcine beta cells. The results obtained using INS-eGFP pig fibroblasts in epigenetic conversion experiments are here presented and discussed. Cells were either plated on plastic or on 1kPa polyacrylamide (PAA) gels -that mimics the stiffness of pancreatic tissue in vivo, erased with 5-aza-CR and exposed to specific pancreatic differentiation stimuli. The use of INS-eGFP fibroblasts, that emit green fluorescence when cells start to produce insulin (thanks to the activation of the GFP linked with insulin construct), allowed real-time monitoring of cell behaviour while transiting along the pancreatic differentiation process in an easy and immediate way (Fig. 3). The results revealed that PAA gels encouraged the induction of islet-like structures, supporting the hypothesis that the formation of tridimensional clusters may be a crucial step of pancreatic differentiation in vitro. Moreover, the use of an adequate substrate accelerated cell differentiation process and anticipated insulin secretion ability. 

## Conclusion

Small molecule ability to modulate cell plasticity and define cell fate can be a powerful tool to induce pluripotency and/or allow phenotype switch of somatic cells. This strategy can be further boosted when cells are plated and grown on an adequate substrate, that displays “*in vivo”* tissue-like stiffness and activates the Hippo signalling mechano-transduction pathway. These findings have interesting impacts in order to ensure an efficient and fast translation process from the laboratory bench to the patient bedside, allowing swift scale-up culture procedures, essential for cell therapy and tissue engineering applied to human regenerative medicine. Moreover, this represents a key point to ensure the development of more physiological models and in turn let for the reduction of the number of animals used, with a particular emphasis on the concept of non-invasive investigations and animal welfare and protection.
